# Identification of glycine betaine as a host-derived molecule required for the vegetative proliferation of the protozoan parasite *Perkinsus olseni*

**DOI:** 10.1017/S0031182023000768

**Published:** 2023-09

**Authors:** Yuqi Liu, Akihiro Ninomiya, Tomoyoshi Yoshinaga, Naoki Itoh

**Affiliations:** 1 Laboratory of Fish Diseases, Graduate School of Agricultural and Life Sciences, The University of Tokyo, Tokyo, Japan; 2 Laboratory of Aquatic Natural Products Chemistry, Graduate School of Agricultural and Life Sciences, The University of Tokyo, Tokyo, Japan

**Keywords:** Betaine aldehyde, biosynthesis, choline, host–parasite interaction, mollusks, Perkinsozoa, schizogony, trimethylglycine, trophozoite

## Abstract

*Perkinsus olseni* is an industrially significant protozoan parasite of Manila clam, *Ruditapes philippinarum*. So far, various media, based on Dulbecco's Modified Eagle Medium and Ham's F-12 nutrient mixture with supplementation of fetal bovine serum (FBS), have been developed to proliferate the parasitizing trophozoite stage of *P. olseni*. The present study showed that *P. olseni* did not proliferate in FBS-deficient Perkinsus broth medium (PBMΔF), but proliferated well in PBMΔF supplemented with tissue extract of host Manila clams, indicating that FBS and Manila clam tissue contained molecule(s) required for *P. olseni* proliferation. Preliminary characterization suggested that the host-derived molecule(s) was a heat-stable molecule(s) with a molecular weight of less than 3 kDa, and finally a single molecule required for the proliferation was purified by high-performance liquid chromatography processes. High-resolution electrospray ionization mass spectrometry and nuclear magnetic resonance analyses identified this molecule as glycine betaine (=trimethylglycine), and the requirement of this molecule for *P. olsseni* proliferation was confirmed by an assay using chemically synthesized, standard glycine betaine. Although glycine betaine was required for the proliferation of all examined *Perkinsus* species, supplementation of glycine betaine precursors, such as choline and betaine aldehyde, enhanced the proliferation of 4 *Perkinsus* species (*P. marinus, P. chesapeaki, P. mediterraneus* and *P. honshuensis*), but not of 2 others (*P. olseni* and *P. beihaiensis*). Thus, it was concluded that the ability to biosynthesise glycine betaine from its precursors varied among *Perkinsus* species, and that *P. olseni* and *P. beihaiensis* lack the ability required to biosynthesize glycine betaine for proliferation.

## Graphical abstract

**Figure:**
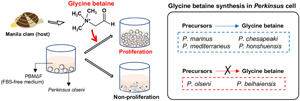

## Introduction

*Perkinsus* is a group of protozoan parasites of marine mollusks, included in the phylum Perkinsozoa, which is now considered to be the earliest group diverging from the lineage leading to dinoflagellates and apicomplexans (Bachvaroff *et al*., 2011; Fernández Robledo *et al*., 2011; Zhang *et al*., 2011). Currently, 7 species have been accepted in this genus: *P. marinus, P. olseni, P. qugwadi, P. chesapeaki, P. mediterraneus, P. honshuensis* and *P. beihaiensis*, and 3 of them (*P. marinus, P. olseni* and *P. qugwadi*) have been accepted as significant pathogens for industrially important mollusks (Azevedo, 1989; Bower *et al*., 1992, 1999; Navas *et al*., 1992; Burreson and Ragone Calvo, 1996; Park and Choi, 2001).

Except for *P. qugwadi*, the other 6 *Perkinsus* species are phylogenetically close to each other, and they have the same life cycle consisting of 4 developmental stages: zoospore, trophozoite, prezoosporangium and zoosporangium (Auzoux-Bordenave *et al*., 1995). The trophozoite-the parasitizing stage within the host tissue-undergoes schizogony by successive bipartitioning of the protoplast to form a schizont containing multiple daughter trophozoites, and these daughter trophozoites are released out when the schizont is ruptured (Mackin *et al*., 1950; Perkins, 1969; Azevedo, 1989; Dungan and Reece, 2006).

*In vitro* culture methods to maintain the schizogony phase and continuous propagation of trophozoites have been developed (Gauthier and Vasta, 1993; La Peyre *et al*., 1993), and currently, media based on Dulbecco's Modified Eagle Medium and Ham's F-12 nutrient mixture (DMEM/Ham's F-12) supplemented with fetal bovine serum (FBS) are widely used (Gauthier and Vasta, 1993, 1995; Dungan and Hamilton, 1995; Gauthier *et al*., 1995).

These *in vitro* culture methods have been used also for studies on the host–parasite interaction of *Perkinsus* species, and various effects of host components (i.e. plasma, tissue lysates and mucus) on *Perkinsus* species, such as modulation of cellular differentiation and infectivity of *P. marinus* and proliferation of *P. mediterraneus*, have been reported so far (Macintyre *et al*., 2003; Earnhart *et al*., 2004; Casas *et al*., 2011; Allam *et al*., 2013). In recent preliminary experiments, *P. olseni* PRA-181 isolated from Manila clam *Ruditapes philippinarum* did not proliferate in the absence of FBS, but proliferated well with supplementation of the tissue extract from host Manila clams (Yang, unpublished), suggesting that clam tissue contains molecule(s) required for *P. olseni* proliferation.

Since the identification of such host-derived molecule(s) is(are) expected to help the understanding of the proliferation mechanisms of this industrially important pathogen, this study aimed to isolate and identify the molecule(s) required for the proliferation of *P. olseni* from Manila clam tissue extracts (CTEs). Additionally, it is highly relevant to determine whether the same molecule(s) is(are) required for the proliferation of congeneric species, as this will help understand the evolution and determination of the host ranges of each species. Thus, this study also aimed to investigate the effects of the identified Manila clam molecule on the proliferation of the other 5 culturable *Perkinsus* species.

## Materials and methods

### Media

In the present study, Perkinsus broth medium (PBM) (ATCC medium 1886) (Table 1), a DMEM/Ham's F-12 derivative medium, was used as the basal medium for the propagation of *Perkinsus* species (Table 2). To examine the proliferation effects of FBS and tissue extract of Manila clams, PBMΔF, a modified PBM without supplementation of FBS, was used. Additionally, to exclude the effect of choline, a glycine betaine precursor, a customized medium PBMΔFΔC was purchased, which did not contain FBS and choline chloride from Gmep Inc. (Fukuoka, Japan). All media were sterilized through 0.22 *μ*m filters, and 2% (v/v) of an antibiotic solution (50 mg mL^−1^ of streptomycin and 50 000 units mL^−1^ of benzylpenicillin potassium) was supplemented before use.

**Table 1.** Composition of Perkinsus broth medium (PBM)

Ingredient	Concentration (g L^−1^)
Daigo's artificial seawater	16
Dulbecco's Modified Eagle's Medium Base (Sigma D5030)	4.2
Nutrient mixture F-12 Ham (Sigma N6760)	5.4
L-Glutamine	0.15
HEPES powder	5.96
NaHCO_3_	0.65
Glucose	0.5
Trehalose	0.1
Galactose	0.1
Lipid concentrate (mL L^−1^)	10
Phenol red (mL L^−1^)	0.5
Fetal bovine serum (mL L^−1^)	20

**Table 2.** Isolates of *Perkinsus* spp. used for the experiments

Species and isolate	Origin		Reference
	Host	location	
*P. marinus* PRA-240	*Crassostrea virginica*	Maryland, USA	Fernández-Robledo *et al*. (2008)
*P. olseni* PRA-181	*Ruditapes philippinarum*	Mie, Japan	Dungan and Reece (2006)
*P. chesapeaki* PRA-66	*Mya arenaria*	Maryland, USA	Reece *et al*. (2008)
*P. mediterraneus* Pmed-G2	*Ostrea edulis*	Menorca, Spain	Casas *et al*. (2008)
*P. honshuensis* PRA-177	*R. philippinarum*	Mie, Japan	Dungan and Reece (2006)
*P. beihaiensis* PRA-431	*Mytilus galloprovincialis*	Kanagawa, Japan	Liu *et al*. (2022)

### Effects of Manila CTE on *Perkinsus olseni* proliferation

#### Manila CTE

Manila clams (mean shell length ± s.d.: 46.51 ± 2.8 mm, *n* = 19) were purchased from the Akkeshi Fisheries Cooperative Association, where the occurrence of *Perkinsus* spp. has not yet been reported. These clams were maintained at 20 °C and 30 practical salinity units in 1-ton circulating aquaria with a biological filter until use.

Gill and mantle tissues of clams were excised with sterile scissors, and the excised tissues were added to the same weight of filter-sterilized half-strength artificial seawater (HASW) made of 18.2 g L^−1^ of Daigo's Artificial Seawater SP for Marine Microalgae Medium (FUJIFILM Wako Pure Chemical Co., Tokyo, Japan) with 2% supplementation of the above antibiotic solution. Then, tissues were manually homogenized with a glass homogenizer on ice, and the homogenate was then transferred to a 50 mL centrifuge tube for centrifugation at 20 000 *g* for 30 min at 4 °C. The supernatant was recovered and transferred to a new centrifuge tube for centrifugation as above, and this step was repeated 2–3 times to obtain clear supernatant. A part of the clear supernatant was used to determine protein concentration using the BCA Protein Assay kit (Takara Bio Inc., Shiga, Japan) according to the manufacturer's protocol, and then the supernatant was filtered through a 0.22 *μ*m filter (AS ONE Co., Osaka, Japan) and stored at −20 °C until use.

#### *In vitro* culture of *P. olseni* with the supplementation of tissue extract

Trophozoites of *P. olseni* PRA-181 were maintained in PBM at 25 °C. Three weeks after the subculture, when trophozoites reached the logarithmic growth phase, trophozoites were harvested by centrifugation at 225 *g* for 5 min. Collected cells were washed twice by resuspending in artificial seawater (ASW) made of 36 g L^−1^ of Daigo's Artificial Seawater SP for Marine Microalgae Medium and centrifugation, and washed cells were resuspended in PBMΔF. The cell suspension was passed through a 27-G needle with a 1-mL syringe more than 5 times for disaggregation, and cell density was evaluated using a Bürker-Türk hemocytometer.

To examine the effects of Manila clam extract on *P. olseni* proliferation, the prepared tissue extract was diluted to adjust protein concentrations at 10, 5, 2.5 and 1 mg mL^−1^ with HASW, and then 3% (v/v) of these dilutions were added to PBMΔF to achieve final protein concentrations of tissue extract at 0.3, 0.15, 0.075 and 0.03 mg mL^−1^, respectively. Then, washed trophozoites suspended in PBMΔF were inoculated into these tissue extract-supplemented media at 5.0 × 10^5^ cells mL^−1^. For the positive and negative controls, the same trophozoite suspension was added to PBMΔF with 2% (v/v) of FBS (namely, PBM) and PBMΔF supplemented with 3% (v/v) of HASW at the same cell density, respectively. Then, 10 mL of all inoculated media were dispensed in 25 cm^2^ flasks and incubated at 25°C. For all experimental groups, 2 biological replicate flasks were prepared.

On 1, 4, 7 and 12 days after incubation, 500 *μ*L of cell suspension of each flask was sampled and cell density was evaluated using a Bürker-Türk hemocytometer after disaggregation by passing the suspension through a 27-G needle. The cell density of each flask was examined twice as technical replicates.

#### Observation of cell morphology of *P. olseni* cells cultured in PBM and PBMΔF

Trophozoites of *P. olseni* were cultured in PBM or PBMΔF for over 2 weeks without changing or adding the medium. Harvested cells were stained with Hoechst 33 342 (Dojindo Laboratories, Kumamoto, Japan) and PlasMem Bright Green (Dojindo Laboratories Co., Ltd., Kumamoto, Japan) for staining nuclei and plasma membrane, respectively, and observed under a microscope equipped with differential interference contrast (DIC) and fluorescence observation (Olympus BX60, Olympus, Tokyo, Japan).

### Characterization of the molecule required for *P. olseni* proliferation

#### Manila CTE and subsequent processing

Manila clams (mean shell length ± s.d.: 44.84 ± 2.9 mm, n = 33) of the same origin as above were purchased, and their gill and mantle tissues were used to prepare tissue extract as above. In the above experiment, the effect on *P. olseni* proliferation was confirmed even when a small amount of tissue extract was added, and thus it was considered that the salinity of the solvent for extraction was negligible. Thus, in this experiment, ASW was used to prepare tissue extract instead of HASW, and this extract was designated as CTE.

To estimate the characteristics of the molecule required for *P. olseni* proliferation, another 2 types of CTE were prepared. Heat-treated extract (CTE-H) was obtained from supernatant by centrifugation (15 000 *g*, 15 min) of CTE (protein concentration 10 mg mL^−1^) heated at 100 °C for 30 min, whereas CTE-H-3000, a fraction containing molecules less than 3 kDa in CTE-H, was prepared by applying CTE-H to a Vivaspin® 20 ultrafiltration unit with a membrane of 3000 MWCO PES and centrifuged at 20 000 *g*, 4 °C, for 8–10 hours. Both CTE-H and CTE-H-3000 were passed through a syringe filter of 0.22 *μ*m pore size and stored at 4 °C until use.

#### *In vitro* culture of *P. olseni* with the supplementation of CTE, CTE-H, and CTE-H-3000

Suspensions of *P. olseni* trophozoites were prepared as above, and then inoculated in PBMΔF supplemented with 0.75% (v/v) of CTE (protein concentration 10 mg mL^−1^), CTE-H, or CTE-H-3000 at a cell density of 2.0 × 10^5^ cells mL^−1^. For the positive and negative controls, trophozoites were inoculated into PBMΔF supplemented with 2% (v/v) of FBS (namely PBM) and PBMΔF supplemented with 0.75% (v/v) of ASW, respectively, at the same cell density. One millilitre of all the inoculated media was dispensed in 3 biological replicate wells of 24-well plates and incubated at 25 °C for 12 days.

As reported by Dungan and Hamilton (1995), to evaluate trophozoite numbers of *P. olseni* and other congeneric species, the usefulness of the tetrazolium-based cell proliferation method was confirmed (Fig. S1), so Cell Counting kit-8 (CCK-8) (Dojindo Laboratories, Tokyo, Japan) was used here to evaluate the proliferation of *P. olseni*. In brief, 100 *μ*L of each inoculated cell suspension in the 24-well plates was transferred to a well of a 96-well plate, and absorbance at 450 nm of each well was measured after the addition of 10 *μ*L of the reaction solution of CCK-8. Each cell suspension in the triplicate 24-well plates was evaluated twice as technical replicates, and the average values were adopted. Since a significant correlation between cell density and absorbance was observed (Fig. S1), cell density was expressed as absorbance at 450 nm in subsequent experiments.

### Isolation and identification of a Manila clam-derived molecule required for *P. olseni* proliferation

Since the presence of a molecule required for *P. olseni* proliferation was confirmed in CTE-H-3000, this fraction was subjected to octadecylsilane (ODS) flash chromatography with methanol and distilled water, which yielded 0, 20, 40, 60, 80 and 100% methanol fractions. The separated fractions were dried *in vacuo* and redissolved in an equal volume of sterile ASW, and then 2% (v/v) of these solutions were added to fresh PBMΔF. *P. olseni* trophozoites suspended in PBMΔF (4.0 × 10^5^ cells mL^−1^) were inoculated in above media containing each fraction in a 1:1 ratio, and 10 mL of the mixtures were transferred into 25 cm^2^ cell culture flasks and incubated at 25°C. Instead of each fraction, ASW and the original extract CTE-H-3000 were added for the negative and positive control, respectively. Three biological replicate flasks were set for each group and incubated for 12 days. The proliferation of *P. olseni* trophozoites was evaluated using the CCK-8 as described above.

The fraction showing the highest effect on *P. olseni* proliferation was further fractionated using an LC-10A HPLC system (Shimadzu, Kyoto, Japan) with a Synergi Polar-RP column (ϕ10 × 250 mm, particle size 4 *μ*m; Phenomenex, Torrance, CA, United States). The mobile phase condition was isocratic elution of 0% methanol for 20 min followed by gradient elution of 0–20% methanol for 10 min, and the final isocratic elution of 100% methanol for 10 min. The eluted compounds were detected by UV absorption at 214 nm, and fractions were dried *in vacuo* and redissolved in sterile ASW to prepare 0.2 mg mL^−1^ solutions, 1% of which were added to fresh PBMΔF (final concentration 2.0 *μ*g mL^−1^ in media). One hundred microlitres of all the inoculated media was dispensed in 3 biological replicate wells of a 96-well plate and incubated at 25 °C for 12 days. Then, the proliferation of *P. olseni* was evaluated using the CCK-8 as described above.

The effective fraction obtained by Synergi Polar-RP was further purified using Asahipak GS-320 HQ column (ϕ7.5 × 300 mm, particle size 6 *μ*m; Showa Denko, Tokyo, Japan) under the mobile phase condition with isocratic elution of 0% methanol for 30 min. The effect of each fraction on *P. olseni* proliferation was evaluated as described above, except that the concentration of each fraction was set at 0.04 mg mL^−1^ (final concentration 0.4 *μ*g mL^−1^ in media).

For molecular identification, the isolated fraction showing the highest effect on *P. olseni* proliferation was subjected to high-resolution electrospray ionization mass spectrometry (HRESIMS) and nuclear magnetic resonance (NMR) analyses. HRESIMS analysis was performed using a TripleTOF 5600 system (SCIEX, Framingham, MA, United States). NMR spectra were recorded on a JNM-ECA600 NMR spectrometer (JEOL Ltd., Tokyo, Japan), and the NMR chemical shifts ^1^H and ^13^C were referenced to the solvent peaks: *δ*_H_ 3.30 and *δ*_C_ 49.0 for CD_3_OD.

Since the target compound was identified to be glycine betaine, the mixture of the chemically synthesized standard glycine betaine (CAS Number: 107-43-7) (FUJIFILM Wako Pure Chemical Co., Tokyo, Japan) and the fraction containing the target compound at a ratio of 1:1 was applied to Asahipak GS-320 HQ column, and its chromatogram was compared to that of standard glycine betaine for further confirmation.

### Effects of glycine betaine on *P. olseni* proliferation

Glycine betaine purified from the host Manila clams (natural glycine betaine) and standard glycine betaine were dissolved in sterile ASW, and 2% (v/v) of these diluted solutions were added to fresh PBMΔF. Final concentrations of glycine betaine in the media were 10, 2 and 0.4 *μ*g mL^−1^ for natural glycine betaine, and 2500, 1000, 100, 10, 2 and 0.4 *μ*g mL^−1^ for standard glycine betaine. For the negative control, 2% (v/v) of sterile ASW was added. One millilitre of all the inoculated media was dispensed in 3 biological replicate wells of 24-well plates and incubated at 25 °C for 12 days. Then, the proliferation of *P. olseni* was evaluated using the CCK-8 as described above. This experiment was conducted in duplicate.

### Effects of glycine betaine and its precursors on the proliferation of *Perkinsus* species

To examine the effects of glycine betaine and its precursors on proliferation, trophozoites of 6 *Perkinsus* species (*P. marinus* PRA-240, *P. olseni* PRA-181, *P. chesapeaki* PRA-66, *P. mediterraneus* Pmed-G2, *P. honshuensis* PRA-177 and *P. beihaiensis* PRA-431; Table 2), were maintained in PBM and harvested at the logarithmic proliferation phase. Since PBMΔF contained the glycine betaine precursor choline, harvested cells were washed and resuspended in FBS- and choline-deficient medium PBMΔFΔC as described above. After evaluating their cell densities with a Bürker-Türk hemocytometer, cell density was appropriately adjusted by fresh PBMΔFΔC.

Suspension of *Perkinsus* spp. trophozoites were inoculated in PBMΔFΔC supplemented with 1.5% (v/v) solutions of the standard glycine betaine or glycine betaine precursors, choline chloride (CAS No. 67-48-1, FUJIFILM Wako Pure Chemical Co., Tokyo, Japan) and betaine aldehyde chloride (Cas No. 7758-31-8, FUJIFILM Wako Pure Chemical Co., Tokyo, Japan). The final concentrations of glycine betaine, choline chloride and betaine aldehyde chloride in media were adjusted at 15, 18 and 15 *μ*g mL^−1^, respectively. The final cell densities inoculated in media were found to be 8.0 × 10^4^ cells mL^−1^ for *P. marinus*, 2.0 × 10^5^ cells mL^−1^ for *P. olseni*, 2.0 × 10^4^ cells mL^−1^ for *P. chesapeaki*, 5.0 × 10^4^ cells mL^−1^ for *P. mediterraneus,* 6.0 × 10^4^ cells mL^−1^ for *P. honshuensis* and 2.0 × 10^5^ cells mL^−1^ for *P. beihaiensis*. For the negative control, the same volume of ASW was added to PBMΔFΔC. One millilitre of all the inoculated media was dispensed in 3 biological replicate wells of 24-well plates and incubated at 25 °C for 12 days. Then the proliferation of each *Perkinsus* species was evaluated using the CCK-8, since the compatibility of this kit for these species was already confirmed (Fig. S1).

### Statistical analysis

The absorbance data from 3 biological replicates and 2 technical replicates were analysed by one-way Analysis of variance (ANOVA) first, then Tukey's post-hoc test was used to compare each pair. Analysis of the results was performed using SPSS (SPSS Inc./IBM, Chicago, Illinois, USA).

## Results

### Effect of tissue extract from host Manila clams at different concentrations on the proliferation of *P. olseni*

**Figure 1.**
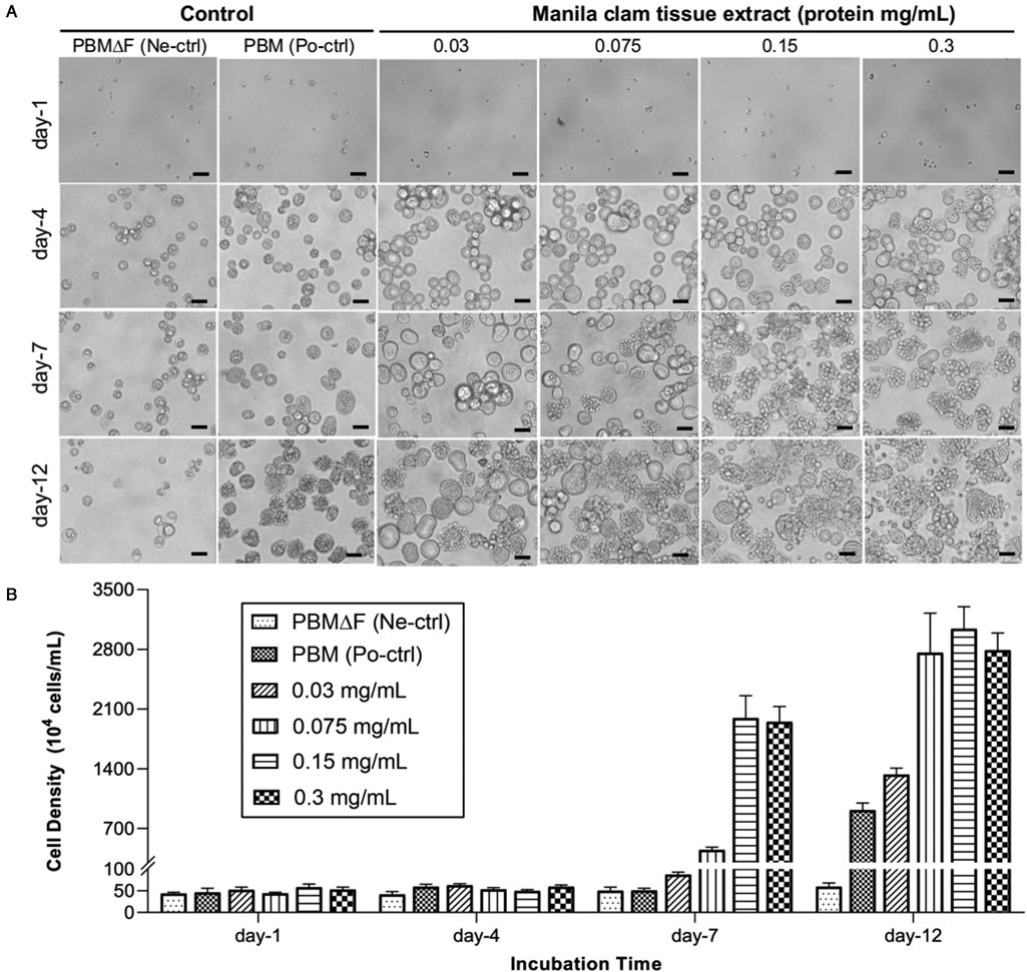
Proliferation of *Perkinsus olseni* inoculated into fetal bovine serum (FBS)-free Perkinsus broth medium (PBMΔF) supplemented with Manila clam tissue extract (CTE) at 4 different concentrations. PBMΔF only and PBMΔF with 2% (v/v) FBS (PBM) were used as the negative (Ne-ctrl) and positive (Po-ctrl) control, respectively. (**A)** Inoculated cells on day-1, day-4, day-7 and day-12 observed by an inverted microscope. Scale bars = 20 *μ*m. **(B)** Cell densities under different concentrations of Manila clam tissue extract (CTE) on day-1, day-4, day-7 and day-12. Bars indicated standard deviation (s.d.) (*n* = 2).

**Figure 2.**
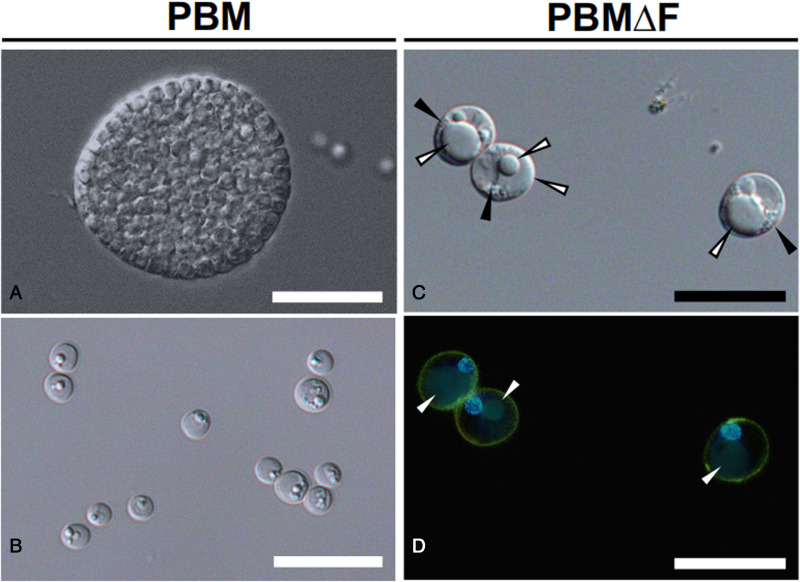
*Perkinsus olseni* inoculated into Perkinsus broth medium (PBM) and fetal bovine serum (FBS)-deficient PBM (PBMΔF) after 2 weeks. **(A)** Mature schizont containing multiple daughter trophozoites and **(B)** daughter trophozoites released from schizont were observed using differential interference contrast microscopy. Nucleus and plasma membranes of trophozoites in PBMΔF were stained with fluorescent dyes Hoechst 33 342 and PlasMem Bright Green respectively and observed using differential interference contrast **(C)** and fluorescent microscopy (**D**, Merge image). White arrowheads: large eccentric refractile vacuoles; black arrowheads: small cytoplasmic refractile granules in cytoplasm matrix. Scale bars = 20 *μ*m.

During incubation in PBM, schizogony processes (enlargement of trophozoites, intracellular formation of daughter cells and release of developed daughter cells) were observed (Figs 1A, 2), and the cell density increased from 4.63 ± 0.94 × 10^5^ at the start of the experiment to 9.15 ± 0.83 × 10^6^ at the end of the experiment on Day 12 (Fig. 1B). On the other hand, *P. olseni* cell density was almost unchanged in PBMΔF during the experiment period, from 4.35 ± 0.31 × 10^5^ to 5.92 ± 0.88 × 10^5^ cells mL^−1^ at the start and end of the experiment on Day 12, respectively (Fig. 1B). Although enlargement of trophozoites was observed, the release of daughter cells was not observed in PBMΔF (Fig. 1A, Figs 2). It is noted that trophozoites in PBMΔF contained large eccentric refractile vacuoles (white arrowhead, Figs 2), and fluorescent stains revealed that these vacuoles were not surrounded by a cell membrane, and were not nuclei (Fig. 2D). In addition, a few smaller cytoplasmic refractile granules were also observed (black arrowhead, Fig. 2C).

In PBMΔF supplemented with the tissue extract, schizogony processes were observed (Fig. 1A), and the cell densities after 12 days of incubation reached 2.79 ± 0.20 × 10^7^, 3.04 ± 0.26 × 10^7^, 2.76 ± 0.46 × 10^7^ and 1.33 ± 0.07 × 10^7^ cells mL^−1^ in groups supplemented with the final protein concentration of the tissue extract at 0.3, 0.15, 0,075 and 0.03 mg mL^−1^, respectively (Fig. 1B).

### Isolation of the host-derived molecule required for proliferation of *P. olseni*

**Figure 3.**
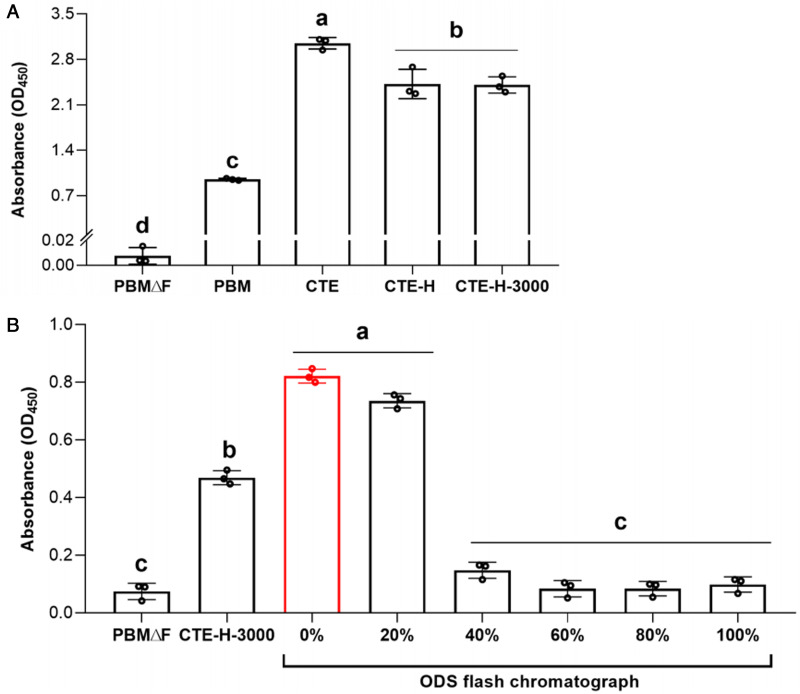
Effect of CTEs after multistep treatments on *Perkinsus olseni* proliferation. **(A)** Cell proliferation of *P. olseni* inoculated in PBMΔF supplemented with Manila clam tissue extract (CTE), heat-treated extract (CTE-H), and a fraction containing molecules less than 3 kDa in CTE-H (CTE-H-3000). PBMΔF and PBM (PBMΔF with 2% (v/v) FBS) were used as the negative and positive control, respectively. **(B)** Cell proliferation of *P. olseni* in PBMΔF supplemented with fractions of CTE-H-3000 fractionated by octadecylsilane (ODS) flash chromatography. PBMΔF and PBMΔF with 1% (v/v) of CTE-H-3000 were used as the negative and positive control, respectively. Red column indicates the fraction showing the highest proliferation effect. Different letters denote statistically significant differences (Tukey's test, *P* < 0.01).

Cell density in PBMΔF supplemented with heated CTE (CTE-H) was significantly lower than unheated CTE, but significantly higher than PBM and PBMΔF (Fig. 3A). Also, cell density in PBMΔF supplemented with CTE-H-3000 was at a similar level to that in CTE-H (Fig. 3A).

Based on the above results, CTE-H-3000 was subjected to ODS flash chromatography, and a higher effect on *P. olseni* proliferation was recognized in 0 and 20% methanol fractions obtained by ODS chromatography, while this effect was not detected in the other fractions (Fig. 3B).

**Figure 4.**
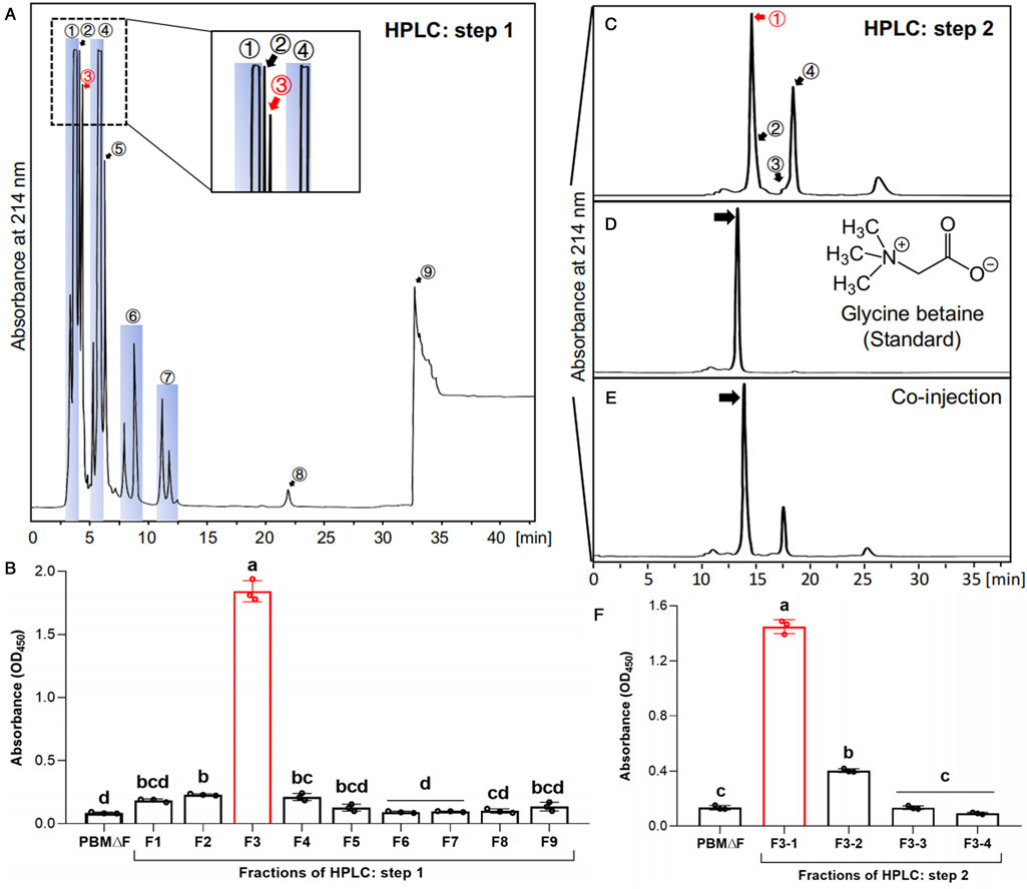
Isolation of Manila clam-derived molecule(s) required for *Perkinsus olseni* proliferation with high-performance liquid chromatograph (HPLC). **(A)** Chromatogram of HPLC using Synergi Polar-RP column for the 0% methanol fraction of ODS flash chromatography. (**B**) Cell proliferation of *P. olseni* in PBMΔF supplemented with 9 fractions obtained from HPLC using Synergi Polar-RP column. **(C)** Chromatogram of HPLC using Asahipak GS-320 HQ column for the third fraction (F3) obtained from Synergi Polar-RP column. **(D-E)** Chromatograms of HPLC using Asahipak GS-320 HQ column for the standard glycine betaine **(D)** and the mixture of the third fraction [F3 obtained from (A)] and standard glycine betaine **(E)**. Arrows indicate glycine betaine. **(F)** Cell proliferation of *P. olseni* in PBMΔF supplemented with 4 fractions obtained from HPLC using Asahipak GS-320 HQ column. Numbers in (A) and (C) correspond to numbers of each fraction in the assay results shown in (B) and (F), respectively, and the blue squares in (A) indicate that components within the region were collected as one fraction. Red arrows in (A) and (C), and red columns in (B) and (F) indicate the fractions showing the highest proliferation effect. Mean values and s.d. of the absorbance are shown (*n* = 3), and different letters denote statistically significant differences (Tukey's test, *P* < 0.01).

The 0% fraction with the highest effect was further fractionated with the Synergi Polar-RP column, and the 3rd fraction of 9 examined fractions (F3, indicated by the red arrow in Fig. 4A) showed the highest effect on *P. olseni* proliferation (Fig. 4B). When this 3rd fraction was further fractionated with the Asahipak GS-320 HQ column, the first fraction (F3-1, indicated by the red arrow in Fig. 4C) showed the highest effect (Fig. 4F), and the compound in this fraction was used for molecular identification.

### Molecular identification

The molecular formula of the target compound in the final F3-1 fraction was determined as C_5_H_11_NO_2_ by HRESIMS (*m/z* 118.0872 [M + H]^+^, calculated for 118.0863). The 1D ^1^H and ^13^C NMR data (Table 3), as well as the 1D and 2D NMR spectra (Figs. S2) showed the presence of 1 carbonyl carbon, 3 equivalent methyl groups, and 1 methylene in this compound, identifying the target compound as glycine betaine.

**Table 3.** ^1^H and ^13^C NMR data

Natural product				Synthetic standard glycine betaine		
Position	*δ*_H_	*δ*_C_	HMBC	Position	*δ*_H_	*δ*_C_
*N*-Me	3.27	53.8	CH_2_	*N*-Me	3.27	53.8
CH_2_	3.82	67.2	*N*-Me, CO	CH_2_	3.82	67.2
CO		168.8		CO		168.8

The NMR data of commercially purchased standard glycine betaine and the final F3-1 fraction were identical to each other (Table 3, Figs. S2). HPLC analyses revealed that the peak of the standard glycine betaine was identical to the peak in co-injection of the fraction F3 and standard glycine betaine, indicating that the major compound in the final F3-1 fraction was glycine betaine (Figs 4D, E).

### Effects of host-derived glycine betaine and standard glycine betaine on the proliferation of *P. olseni*

**Figure 5.**
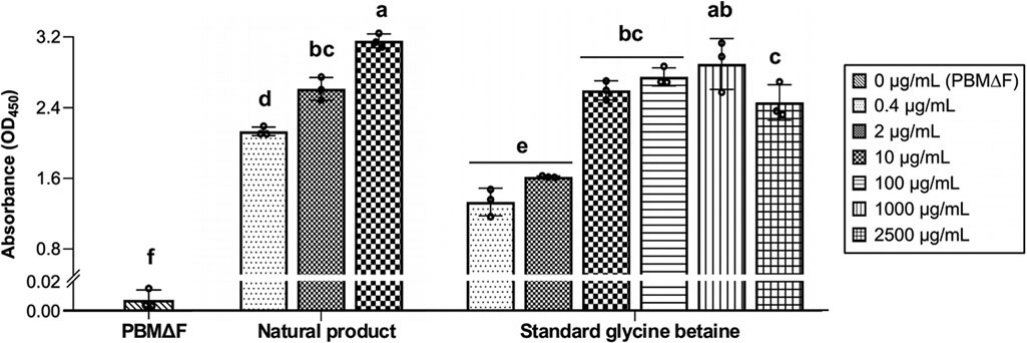
Effect of the host-derived glycine betaine and standard glycine betaine on *Perkinsus olseni* proliferation. Cell proliferation of *P. olseni* in PBMΔF only and PBMΔF supplemented with natural product and commercially purchased standard glycine betaine at different concentrations. Mean values and s.d. of the absorbance are shown (*n* = 3), and different letters denote statistically significant differences (Tukey's test, *P* < 0.01).

Under the presence of either the host-derived glycine betaine or the standard glycine betaine, *P. olseni* showed significantly higher proliferation than that in PBMΔF (Fig. 5), though proliferation under the presence of the host-derived glycine betaine was higher than the standard glycine betaine for the same concentrations (Fig. 5). The effect on *P. olseni* proliferation showed concentration dependence, while this effect declined at 2500 *μ*g mL^−1^ of the standard glycine betaine (Fig. 5).

### Effects of glycine betaine and 2 glycine betaine precursors, choline, and betaine aldehyde, on the proliferation of *Perkinsus* spp.

To investigate glycine betaine requirements and abilities to biosynthesize glycine betaine from the major precursor choline, the proliferation of 6 *Perkinsus* species were examined in PBMΔFΔC supplemented with choline, betaine aldehyde (the intermediate product) or glycine betaine.

**Figure 6.**
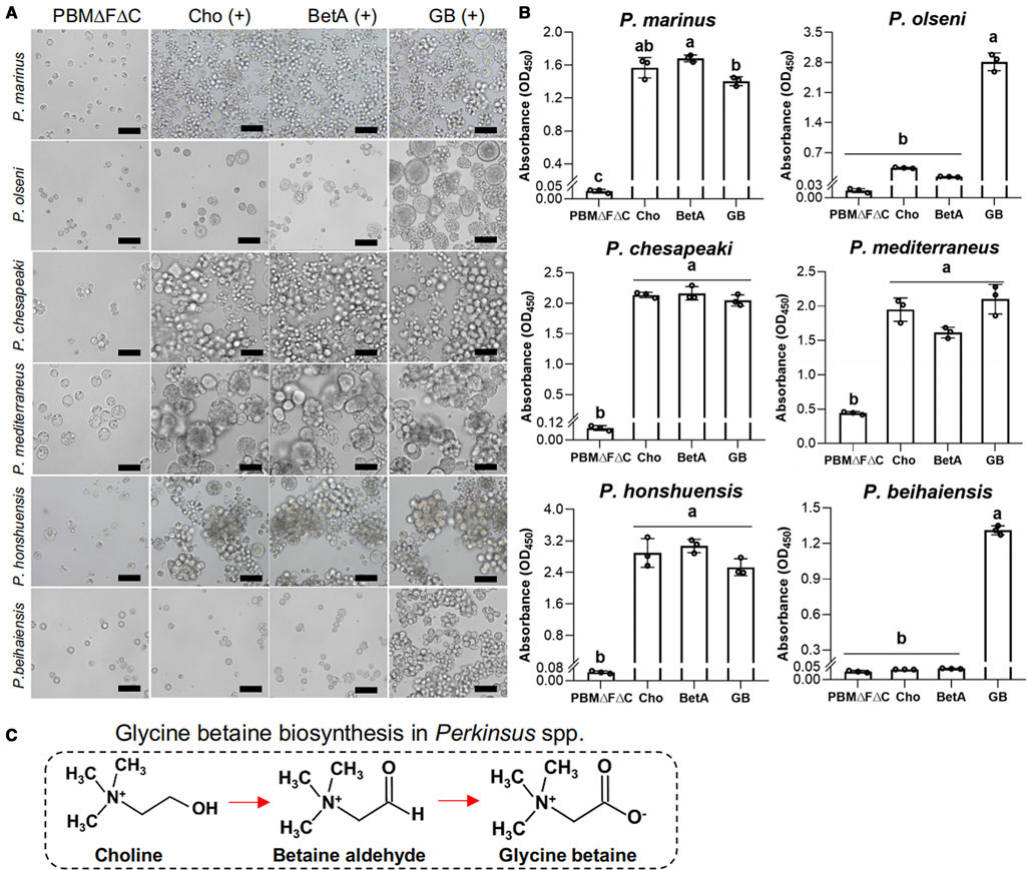
Proliferation of 6 *Perkinsus* spp. in fetal bovine serum, choline-deficient medium PBMΔFΔC supplemented with glycine betaine and its precursors, choline and betaine aldehyde. (**A)** Trophozoites of *Perkinsus* spp. in PBMΔFΔC supplemented with glycine betaine (GB) and its precursors, choline (Cho) and betaine aldehyde (BetA) on day-12. Scale bars = 40 *μ*m. (**B)** Cell densities of 6 *Perkinsus* species. Mean values and s.d. of the absorbance are shown (*n* = 3), and different letters denote statistically significant differences (Tukey's test, *P* < 0.01). (**C)** Synthetic pathway of glycine betaine from choline through betaine aldehyde.

Microscopically, cell proliferation was not confirmed in all 6 examined *Perkinsus* species in PBMΔFΔC without any supplementation until the end of the experiment on Day 12 (Fig. 6A). On the other hand, active proliferation of all examined *Perkinsus* species was observed in PBMΔFΔC supplemented with glycine betaine (Fig. 6A). Supplementation of choline or betaine aldehyde enhanced the proliferation of *P. marinus*, *P. chesapeaki*, *P. mediterraneus* and *P. honshuensis*; however, supplementation of these glycine betaine precursors did not enhance the proliferation of *P. olseni* and *P. beihaiensis* (Fig. 6A).

Similarly, CCK-8 assays also revealed that the proliferation of *P. marinus*, *P. chesapeaki*, *P. mediterraneus* and *P. honshuensis* were significantly higher under the presence of choline, betaine aldehyde and glycine betaine than the negative control of PBMΔFΔC, whereas the proliferation of *P. olseni* and *P. beihaiensis* were higher only in the presence of glycine betaine (Fig. 6B).

## Discussion

The present study revealed that *P. olseni* did not proliferate in FBS-deficient PBM (PBMΔF), but did proliferate in PBMΔF supplemented with the tissue extract of the host Manila clam. HPLC analyses combined with the proliferation assay showed that glycine betaine in the tissue extract was required for the proliferation of *P. olseni*, and this requirement was confirmed using standard glycine betaine. In the presence of glycine betaine precursors, either choline or betaine aldehyde, 4 *Perkinsus* species (*P. marinus*, *P. chesapeaki*, *P. mediterraneus* and *P. honshuensis*) proliferated well without the addition of glycine betaine. On the other hand, *P. olseni* and *P. beihaiensis* proliferated only in the presence of glycine betaine, indicating that *P. olseni* and *P. beihaiensis* did not have the ability to biosynthesize glycine betaine from either precursors, unlike the other 4 species.

For *in vitro* culture of *P. olseni*, various media based on DMEM:Ham's F-12 supplemented with FBS have been widely applied (Ordás and Figueras, 1998; Coss *et al*., 2001; Dungan *et al*., 2007). In the present study, *P. olseni* trophozoites in the FBS-deficient medium PBMΔF became enlarged with large eccentric vacuoles but did not develop into schizonts, and the results of fluorescent staining confirmed that the cells in PBMΔF were single cells without nuclear division (Figs 2), indicating that FBS contained molecule(s) required for the proliferation of *P. olseni*. Interestingly, under the presence of Manila CTE, *P. olseni* proliferated rapidly even in FBS-deficient PBMΔF, suggesting that Manila clam contains molecule(s) necessary for *P. olseni* proliferation.

To date, several studies have been conducted to examine the effects of host-derived substances on the proliferation of *Perkinsus* spp. It has been reported that the supplementation of the host oyster lysate promotes the proliferation of *P. mediterraneus* (Casas *et al*., 2011). On the other hand, the pallial-organ mucus of the eastern oyster rapidly and significantly promotes *P. marinus* cell proliferation (Allam *et al*., 2013; Espinosa *et al*., 2013), while plasma and whole soft tissue extracts have been found to inhibit *P. marinus* growth (MacIntyre *et al*., 2003; Earnhart *et al*., 2004). Hence, the effects of host's composition on the proliferation of *Perkinsus* spp. may vary depending on the *Perkinsus* species, host species and the origin of the tissue extracts.

The present study showed that the Manila clam-derived molecule(s) required for *P. olseni* proliferation was a heat-stable small molecule(s) with a molecular weight of less than 3 kDa (Fig. 3A). As this molecule(s) was limited to the CTE-H-3000 fraction, purification processes of this extract were followed to identify the molecule(s). Although the possibility was not ruled out that there were multiple host-derived molecules required for the trophozoite proliferation, we used the single fraction showing the highest effect on *P. olseni* proliferation for the subsequent HPLC purification process, and finally, the single responsible molecule was isolated (Figs 4).

NMR and HRESIMS analyses indicated only 1 possible structure for the molecule, and the putative host-derived molecule was identified as trimethylglycine, also known as glycine betaine. It is worth noting that there was a slight delay between the retention time of clam-derived natural glycine betaine (Fig. 4C) and standard glycine betaine using Asahipak GS-320 HQ column (Fig. 4D). However, a similar effect on *P. olseni* proliferation was confirmed in PBMΔF supplemented with natural and standard glycine betaine (Fig. 5). Thus, the observed delay may be caused by the presence of other impurities in the fraction containing the clam-derived natural glycine betaine, and it was concluded that glycine betaine is a host-derived molecule required for *P. olseni* proliferation. The presence of glycine betaine is also known in fetal calf serum (Lever *et al*., 1994), which is almost the same composition as FBS, strongly indicating that the reason to supplement FBS for *P. olseni* propagation is to supply glycine betaine. It is noted that 1 strain of *P. olseni* originated in a clam species, *Tapes decussatus* of Europe, was successfully proliferated in an FBS-free medium, JL-ODRP-2A (La Peyre *et al*., 1995; Casas *et al*., 2002). However, one of the medium compositions, yesastolate contains glycine betaine (Sakaguchi, 1960), and yeastolate in JL-ODRP-2A should be a source of glycine betaine for the proliferation of *P. olseni* instead of FBS.

Glycine betaine is an amino acid derivative originally found in sugar beet (Scheibler, 1869). Currently, most eukaryotic organisms are known to have the ability to biosynthesize glycine betaine by a 2-step oxidization of choline through the intermediate product betaine aldehyde (Fig. 6C) (Annunziata *et al*., 2019), and glycine betaine has been widely detected in microorganisms, animals and plants (Blunden *et al*., 1996, 2003; Adrian-Romero *et al*., 1998). Glycine betaine is known to play a role of an osmolyte modulator, but also functions as a methyl donor participating in the methionine-homocysteine cycle for various biochemical reactions, such as DNA methylation (Kettunen *et al*., 2001; Lever and Slow, 2010; Zeisel, 2017).

Given the requirement of glycine betaine supplementation for *P. olseni* proliferation, it was considered that *P. olseni* without the ability to biosynthesize glycine betaine would not proliferate because DNA methylation, which is essential for trophozoite proliferation, was inhibited under the absence of glycine betaine. However, even though PBMΔF and PBMΔFΔC used in this study contained methionine as a basal component, the proliferation of *P. olseni* was not observed. Additionally, supplementation of methionine in these media did not enhance the proliferation of *P. olseni* (data not shown). Therefore, it is likely that glycine betaine may be involved in the proliferation of *P. olseni* in ways other than DNA methylation. Recently, the involvement of glycine betaine in various intracellular signalling pathways has been proposed, such as stress-mediated apoptosis (Kar *et al*., 2019), regulation of AKT/ ERK1/2/p38 MAPK pathways (Li *et al*., 2019), and p53-induced apoptosis (Guo *et al*., 2015), although details of these mechanisms remain unknown. In the future, *P. olseni* may become a model organism to discover novel intracellular signalling pathways involving glycine betaine.

It has been known that Manila clam has as high as 4.1 mg g^−1^ tissues of glycine betaine concentration (Farabegoli *et al*., 2019), and other mollusk species are also known to contain relatively high concentrations of glycine betaine (Konosu and Hayashi, 1975; Wright *et al*., 1992; Pierce *et al*., 1995; de Zwart *et al*., 2003). Therefore, it is interesting whether *Perkinsus* species, specifically those parasitizing mollusks, commonly lose the ability to biosynthesize glycine betaine.

Then, using PBMΔFΔC which did not contain FBS and the glycine betaine precursors choline and betaine aldehyde, the effects of glycine betaine and glycine betaine precursors on *Perkinsus* proliferation were examined. No examined *Perkinsus* species proliferated in PBMΔFΔC, but all actively did when supplemented with glycine betaine (Figs 6A, B), indicating glycine betaine was required for the proliferation of all 6 *Perkinsus* parasites. However, 4 species, *P. marinus*, *P. chesapeaki, P. mediterraneus* and *P. honshuensis,* proliferated actively under the presence of glycine betaine precursors, choline and betaine aldehyde, without glycine betaine supplementation, suggesting that these 4 species have the ability to biosynthesize glycine betaine for proliferation from these precursors (Fig. 6C). This consideration was also supported by the active proliferation of these 4 species in PBMΔF containing choline as a basic composition (Figs. S3). Various culture media incorporating DMEM and HAM's F-12 with FBS supplementation have been developed for *Perkinsus* species (Gauthier and Vasta, 1993, 1995). However, since DMEM and HAM's F-12 already contain choline chloride, FBS supplementation may not be necessary for these 4 species. On the other hand, supplementation of glycine betaine precursors only did not lead to proliferation of *P. olseni* or *P. beihaiensis*, similar to the negative control of PBMΔFΔC (Figs 6A, B), demonstrating that only *P. olseni* and *P. beihaiensis* lack the ability to biosynthesize glycine betaine from its precursors (Fig. 6C).

*P. olseni* and *P. beihaiensis* not only lack the ability to synthesize glycine betaine (present study), they also have a broader range of host species and wider geographic distribution than the other species of *Perkinsus* (Villalba *et al*., 2004; Pagenkopp Lohan *et al*., 2016, 2018; Liu *et al*., 2022), so they may have evolutionally acquired some advantages to expand host species and geographic distribution by losing the ability to biosynthesize glycine betaine. However, since the present study examined only 1 strain of each *Perkinsus* species, it is not possible to conclude that the observed glycine betaine requirement is a unique feature of each species. In fact, Gauthier and Vasta (1995) found that supplementation with FBS positively affected the proliferation of *P. marinus*, suggesting possible variations in glycine betaine biosynthetic capability among strains of the same species. In the future, further studies on the glycine betaine requirement of each species should be re-examined for a large number of strains from different geographic locations and host species.

## Supplementary material

For supplementary material accompanying this paper visit https://doi.org/10.1017/S0031182023000768. click here to view supplementary material
