# Supplementary material for: Identification of glycine betaine as a host-derived molecule required for the vegetative proliferation of the protozoan parasite Perkinsus olseni
Source: Parasitology. 2023 Aug 11;150(10):939–49. doi: 10.1017/S0031182023000768 (PMC10577664; doi:10.1017/S0031182023000768)
Supplement: Supplementary file 1 [file S0031182023000768sup.zip › S0031182023000768sup001.docx]

**Supplementary Fig S1. Evaluation of *Perkinsus* spp. trophozoite density using a tetrazolium-based cell proliferation kit**


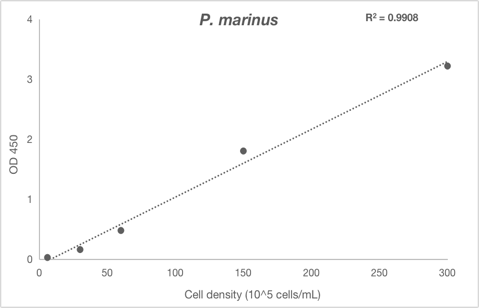

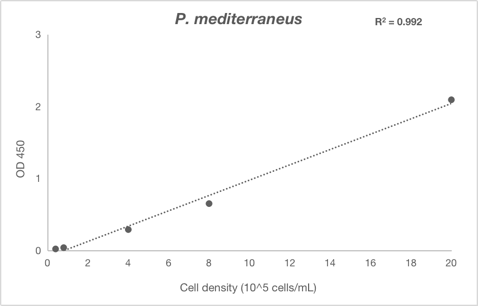

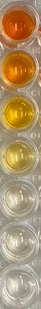

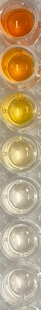

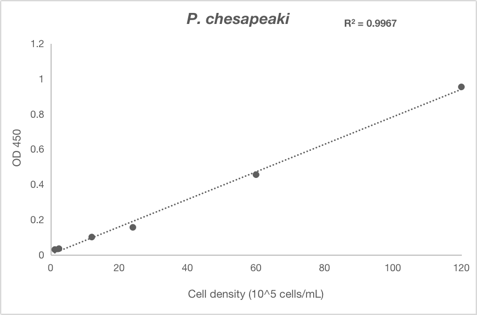

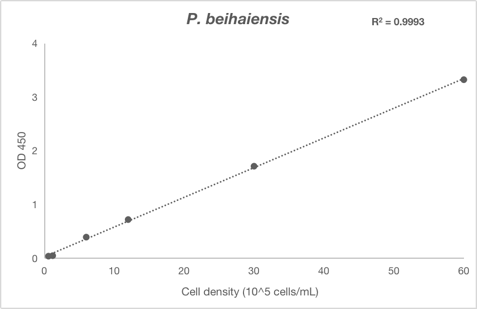

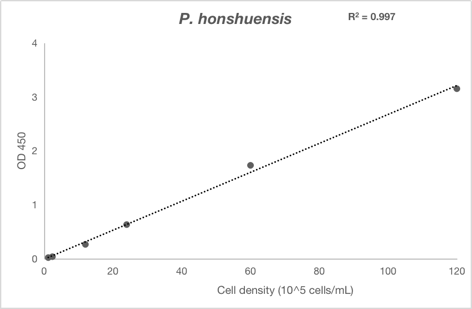

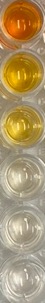

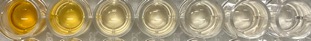

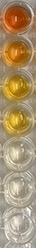

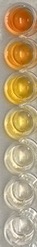

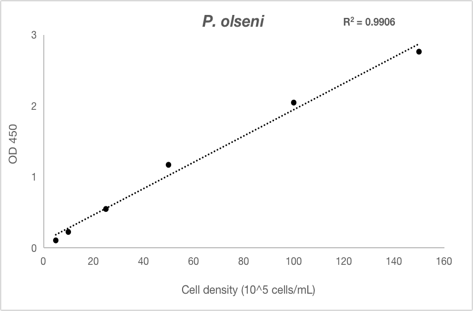


Cell suspensions of 6 *Perkinsus* trophozoites (Table 2) in the logarithmic phase were serially diluted with fresh PBM∆F, and 100 µL of each cell density suspension was inoculated into three wells of a 96-well cell culture plate. Ten microliters of the reaction solution of the Cell Counting kit-8 (Dojindo Laboratories, Kumamoto, Japan) was dispensed into each well, and the plate was incubated at 25 °C for 3-4 hours. Then, the absorbance of each well was measured at 450 nm using a microplate reader (model MRP-A4, Tosoh, Tokyo, Japan). The obtained data revealed that absorbances at 450 nm were positively correlated with trophozoite densities of all examined *Perkinsus* species.
