# Supplementary material for: Identification of glycine betaine as a host-derived molecule required for the vegetative proliferation of the protozoan parasite Perkinsus olseni
Source: Parasitology. 2023 Aug 11;150(10):939–49. doi: 10.1017/S0031182023000768 (PMC10577664; doi:10.1017/S0031182023000768)
Supplement: Supplementary file 1 [file S0031182023000768sup.zip › S0031182023000768sup002.docx]

**Supplementary Fig S2. NMR spectra of the target product and standard glycine betaine.**

***A***^1^H-NMR (above) and ***B*** ^13^C-NMR (below) spectra of the target product

**A**

**B**

***C*** HMQC (above) and ***D*** HMBC (below) spectra of the target compound

**D**

**C**

***E*** ^1^H-NMR (above) and ***F*** ^13^C-NMR (below) spectra of the standard glycine betaine.

**F**

**E**
