# Supplementary material for: Identification of glycine betaine as a host-derived molecule required for the vegetative proliferation of the protozoan parasite Perkinsus olseni
Source: Parasitology. 2023 Aug 11;150(10):939–49. doi: 10.1017/S0031182023000768 (PMC10577664; doi:10.1017/S0031182023000768)
Supplement: Supplementary file 1 [file S0031182023000768sup.zip › S0031182023000768sup003.docx]

**Supplementary Fig S3.** **Proliferation of *Perkinsus* spp. in PBM and PBM∆F**


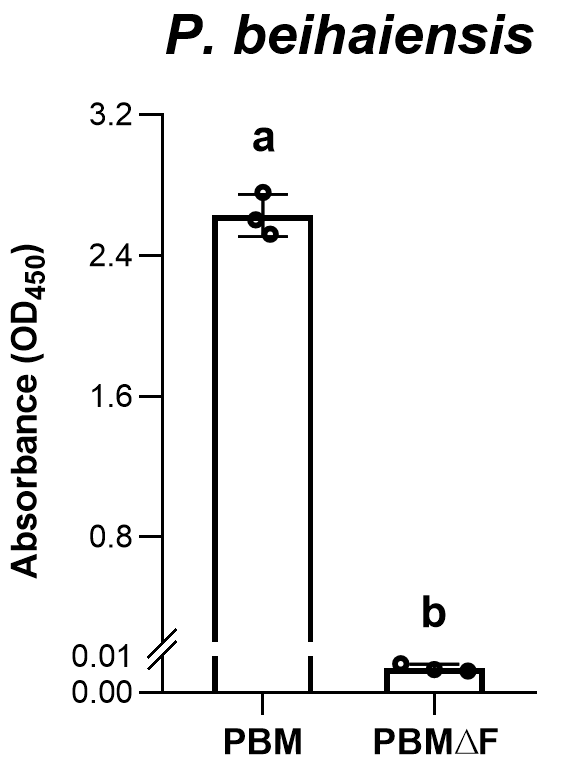

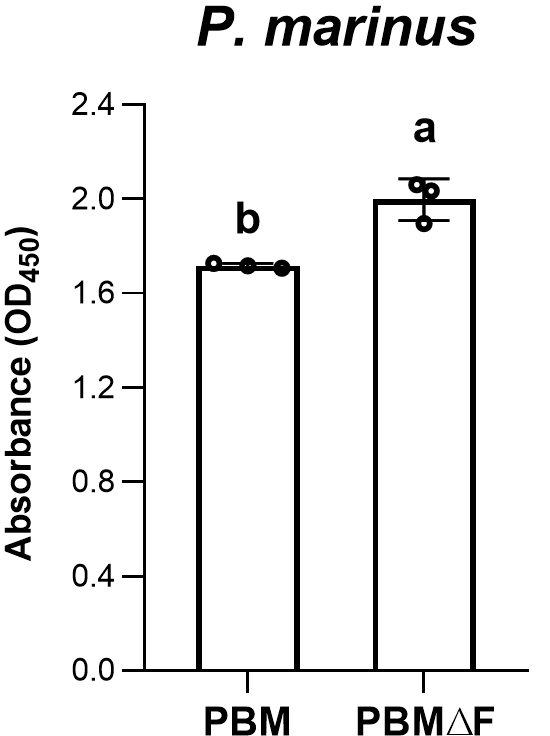

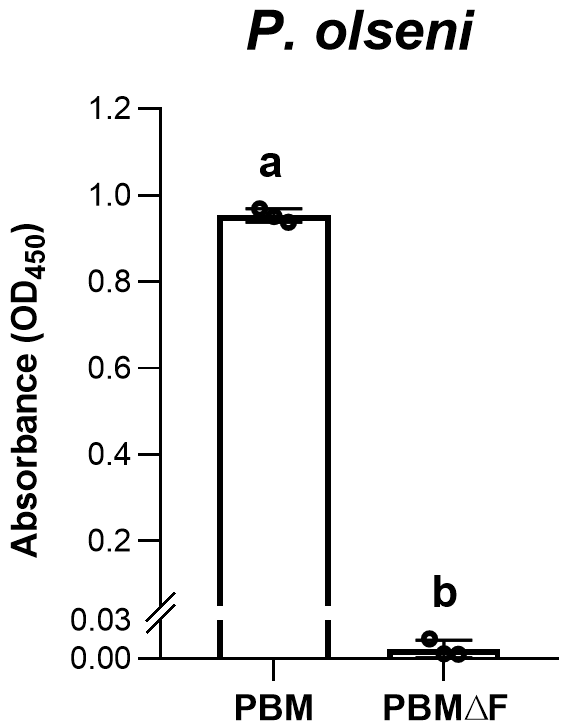

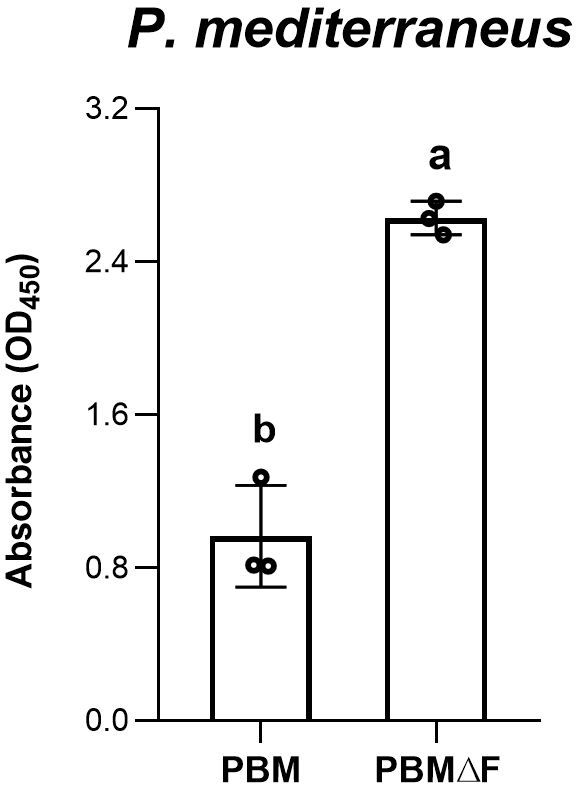

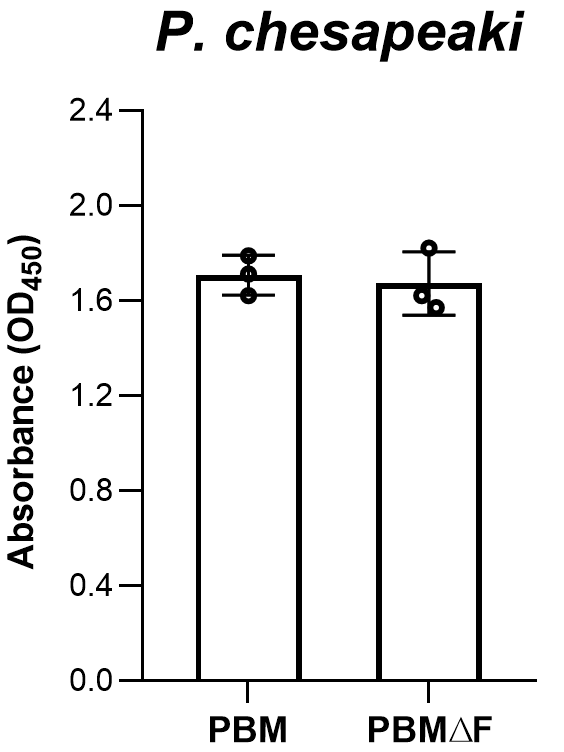

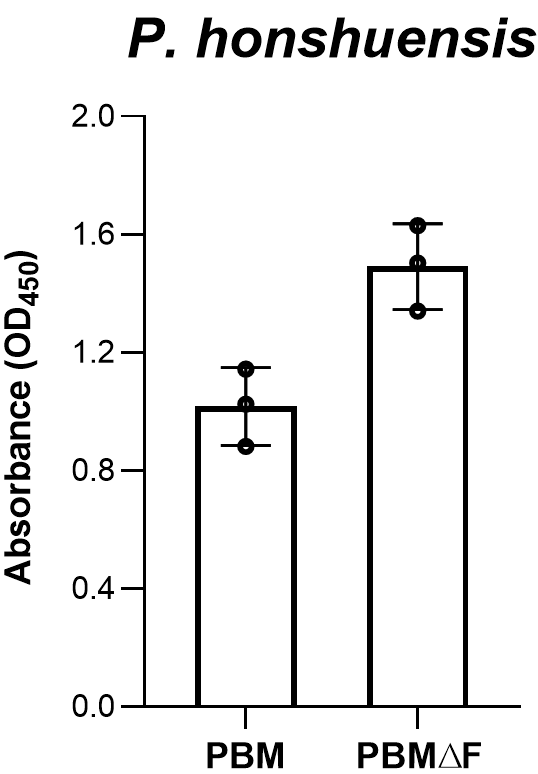


Trophozoites of 6 *Perkinsus* species [*P. marinus* PRA-240, *P. olseni* PRA-181, *P. chesapeaki* PRA-66, *P. mediterraneus* Pmed-G2, *P. honshuensis* PRA-177, and *P. beihaiensis* PRA-431 (Table 2)] were maintained in PBM and harvested at the logarithmic proliferation phase. Harvested cells were washed and resuspended in PBM∆F, and cell densities were counted by a Bürker-Türk hemocytometer. Trophozoites of each species were inoculated into fresh PBM∆F and PBM∆F supplemented with 2% (v/v) FBS (namely PBM), at the final cell density in media of 8.0 × 10^4^ cells/mL for *P. marinus*, 2.0 × 10^5^ cells/mL for *P. olseni*, 2.0 × 10^4^ cells/mL for *P. chesapeaki*, 5.0 × 10^4^ cells/mL for *P. mediterraneus*, 6.0 × 10^4^ cells/mL for *P. honshuensis*, and 2.0 × 10^5^ cells/mL for *P. beihaiensis*. Triplicate wells in a 24-well plate were prepared for each group, and the proliferation, expressed as ABS at 450 nm, was evaluated using the CCK-8 (Dojindo Laboratory, Kumamoto, Japan) after 12 days of incubation. Statistical analyses were conducted by Tukey’s post-hoc test, and different letters denote statistically significant differences (*p* < 0.01).
